# Fabrication of bone‐derived decellularized extracellular matrix/ceramic‐based biocomposites and their osteo/odontogenic differentiation ability for dentin regeneration

**DOI:** 10.1002/btm2.10317

**Published:** 2022-04-05

**Authors:** Dongyun Kim, Hyeongjin Lee, Geum‐Hwa Lee, The‐Hiep Hoang, Hyung‐Ryong Kim, Geun Hyung Kim

**Affiliations:** ^1^ Department of Biomechatronic Engineering, College of Biotechnology and Bioengineering Sungkyunkwan University (SKKU) Suwon Republic of Korea; ^2^ Non‐Clinical Evaluation Center, Biomedical Research Institute Jeonbuk National University Hospital Jeonju Republic of Korea; ^3^ Department of Pharmacology, College of Dentistry Jeonbuk National University Jeonju Republic of Korea; ^4^ Biomedical Institute for Convergence at SKKU (BICS) Sungkyunkwan University Suwon Republic of Korea

**Keywords:** 3D bioprinting, biocomposite, bone regeneration, decellularized extracellular matrix, dentin regeneration

## Abstract

The goal of this study was to fabricate bioactive cell‐laden biocomposites supplemented with bone‐derived decellularized extracellular matrix (dECM) with calcium phosphate ceramic, and to assess the effect of the biocomponents on the osteogenic and odontogenic differentiation of human dental pulp stem cells (hDPSCs). By evaluating the rheological properties and selecting printing parameters, mechanically stable cell‐laden 3D biocomposites with high initial cell‐viability (>90%) and reasonable printability (≈0.9) were manufactured. The cytotoxicity of the biocomposites was evaluated via MTT assay and nuclei/F‐actin fluorescent analyses, while the osteo/odontogenic differentiation of the hDPSCs was assessed using histological and immunofluorescent analyses and various gene expressions. Alkaline phosphate activity and alizarin red staining results indicate that the dECM‐based biocomposites exhibit significantly higher osteogenic activities, including calcification, compared to the collagen‐based biocomposites. Furthermore, immunofluorescence images and gene expressions demonstrated upregulation of dentin matrix acidic phosphoprotein‐1 and dentin sialophosphoprotein in the dECM‐based biocomposites, indicating acceleration of the odontogenic differentiation of hDPSCs in the printed biocomposites. The hDPSC‐laden biocomposite was implanted into the subcutaneous region of mice, and the biocomposite afforded clear induction of osteo/odontogenic ectopic hard tissue formation 8 weeks post‐transplantation. From these results, we suggest that the hDPSC‐laden biocomposite is a promising biomaterial for dental tissue engineering.

## 1 INTRODUCTION

Biophysically and biochemically functionalized three‐dimensional (3D) constructs have become critical for the successful regeneration of various tissues.^1^, ^2^ In particular, tissue engineering strategies, including cell‐based regeneration using dental pulp stem cells (DPSCs) and scaffolds combined with bioactive growth factors (i.e., basic fibroblast growth factor (bFGF), epidermal growth factor (EGF), and bone morphogenic protein (BMP)).^3^, ^4^, ^5^ have been used to successfully regenerate intricate dental tissues; nevertheless, new bioactive tissue‐regenerating materials dedicated to dental tissues are still being investigated.^6^, ^7^, ^8^, ^9^

3D bioprinting has been extensively applied to fabricate cell‐laden structures using a mixture of cells and bioactive hydrogels, which can be termed as a bioink. Because this process can enable the stacking of microscale cell‐laden struts according to a designed 3D structure, the printing system has been considered as an outstanding tool to attain tissue engineering substitutes. Recently, to successfully regenerate various tissues, such as skeletal muscles^10^ and those of the heart,^11^ liver,^12^ and bone,^13^ cell‐affordable, biocompatible, and printable hydrogels, including alginate, gelatin, gelatin methacrylate, silk fibroin, collagen, and methacrylated collagen, have been extensively investigated.^14^, ^15^, ^16^, ^17^, ^18^, ^19^, ^20^ In general, tissue‐specific bioinks to provoke chosen cellular activities can help induce the growth and differentiation of laden cells. Specific microcellular environmental conditions can also be attained with supplementary bioactive components (such as growth factors, RGD ligands, and cytokines) physically or chemically bound in the hydrogels. The developed bioinks provide outstanding regeneration ability of various tissues, but they cannot completely demonstrate the biochemical intricacies of the natural tissue‐specific extracellular matrix (ECM).

In particular, decellularized ECMs (dECMs) derived from bovine bone and dentin have been used as a constituting component of bioinks for 3D dentistry constructs, and the fabricated 3D structures represented reasonable osteo/odontogenic differentiation of the laden cells (DPSCs or odontoblast‐like cell line).^4^, ^21^ However, the regeneration of odontogenic tissues to mimic the organic/inorganic compounds of a native dentin construct is challenging.

Recently, alginate‐ or Matrigel‐based bioinks supplemented with various bioceramics (such as α‐tricalcium phosphate [α‐TCP], nano‐hydroxyapatite, and biphasic calcium phosphate) were constructed using a molding and printing process to overcome the weak mechanical nature of hydrogels and improve the osteoinductive properties of the bioinks.^22^, ^23^, ^24^ In these studies, viable cells resided well within the structures; however, the potential for in vitro osteogenic activity and in vivo new bone formation and angiogenesis analysis using stem cells have not been fully investigated. Previously, we developed a bioink containing collagen and α‐TCP using human adipose stem cells (hASCs) for bone tissue regeneration.^25^ We focused on the in vitro osteogenic differentiation of hASCs loaded in the composite bioink with and without osteogenic medium.^25^ Our work showed a significant potential for osteogenic differentiation lineage of the hASCs when using the bioink containing bioceramic, but the study was limited in terms of the degree of osteogenic activities of the hASCs loaded in the collagen/ceramic‐bioink with and without an osteogenic medium.

Here, we utilized the bioprinting process with a dental‐specific bioink containing hDPSCs to manufacture biomimetic 3D dental constructs. To accomplish this, collagen type‐I or dECM derived from porcine bone as matrix hydrogels of the cells were physically mixed with an appropriate concentration of β‐TCP for the fabrication of a cell‐laden biocomposite. We assumed that the tissue‐specific biochemical cues from the dECM and osteoinductive β‐TCP could synergistically effect cell growth and osteo/odontogenic differentiation of the hDPSCs in the printed dental construct. Based on the rheological properties and by assessing printability, we could construct a 3D cell‐laden mesh scaffold. In vitro biological evaluations (cell viability and growth, calcified tissue matrix, and osteo/odontogenic gene expression) were performed to validate the printed biocomposite with a control, a hDPSC‐laden collagen/β‐TCP bioink. Subsequently, the hDPSC‐laden biocomposite was implanted into the subcutaneous tissue of mice to show that the structure can clearly induce osteo/odontogenic ectopic hard tissue formation 8 weeks after transplantation.

## 2 MATERIALS AND METHODS

### 2.1 Preparation of decellularized extracellular matrixes (ECMs) from porcine bone and dentin tissues

Porcine bone tissue was extracted from the shins and thighs of the fore and hind limbs of a pig. To remove blood and impurities (fibrous tissue and adipose tissue), the bone tissues were placed in a container with deionized water and washed at 120 rpm for 30 min. This was repeated six times. The bone pieces were then crushed using a grinder to obtain bone powder. For demineralization, 0.5 M HCl (Sigma‐Aldrich) was added to the bone powder and stirred for 5 h via magnetic stirring. The stirred solution was sieved using a 100‐μm sieve. The remaining solution was removed via spin‐down using a centrifuge and washed several times with distilled water. A 1:1 ratio of a mixed solution of chloroform (Sigma‐Aldrich) and methanol (Sigma‐Aldrich) was used to remove lipids from the desalted powder, which was washed repeatedly with methanol and then distilled water for 1 h. The demineralized bone matrix (DBM) was obtained via lyophilization. A detailed decellularization protocol was used, as in a previous study.^26^ The DBMs were briefly washed with distilled water and treated with 0.05% trypsin and 0.02% ethylenediaminetetraacetic acid (EDTA, Sigma‐Aldrich) at 37°C for 2 h. Subsequently, the DBMs were treated with 1% w/v penicillin/streptomycin at 4 °C for 24 h to remove residual cellular material and then lyophilized to obtain a dECM. 0.01 M HCl (Sigma‐Aldrich) with pepsin (Sigma‐Aldrich) was added at the density of 1 mg/mL under continuous magnetic stirring for 3 days; 15% w/v of NaCl (Sigma‐Aldrich) was also used for the process. Various ECM proteins were centrifuged, and the remnant components were dialyzed using a dialysis sack (molecular weight cutoff: 3.5 kDa; Spectrum Labs). Following completion of the lyophilization procedure, the dECM was obtained.

To obtain the dentin‐derived dECM, molar teeth were extracted from a pig's jaw and washed several times with Dulbecco's phosphate buffered saline (Biowest, MO, USA), and the enamel layer was removed completely using a sawing process. Subsequently, demineralization and decellularization procedures were performed, identical to the previous protocol for the bone‐derived dECM.

### 2.2 Characterization of decellularized ECMs (dECMs)

To measure the cellularity of native tissue and the dECMs, double‐stranded DNA content was evaluated using the Quant‐iT Picogreen® dsDNA assay kit (Life Technologies). The samples (10 mg/ml) were dissolved in a TE buffer solution (pH 7.5, 10 mM Tris–HCl, and 1 mM EDTA), and then Quant‐iT Picogreen®reagent was added. The mixture was incubated for 5 min at 28°C. A CytoFluor microplate reader (MTX Lab Systems Inc., Vienna, VA) was used to measure absorbance (520 nm).

To quantify the dECM constituents, the amounts of collagen and glycosaminoglycans (GAGs) were assessed. The solubilized collagen, extracted with 0.5 M acetic acid/pepsin at 4°C, was quantified using the Sircol Soluble Collagen Assay (Biocolor Ltd.). The collagen content was determined using a spectrophotometer at 555 nm. Sulfated GAG (sGAG), which was obtained using a papin/pepsin digestion buffer for 3 h at 65°C, was quantified using the Blyscan sGAG Assay (Biocolor Ltd.) at 656 nm.

Quantibody® Human growth factor Array Q1 (RayBiotech) was used to test the concentrations of 40 growth factors in the bone‐derived dECM and dentin‐derived dECM, specifically amphiregulin, brain‐derived neurotrophic factor, bFGF, BMP‐4, BMP‐5, BMP‐7, beta‐nerve growth factor (β‐NGF), EGF, endocrine gland‐derived vascular endothelial growth factor (EG‐VEGF), FGF, growth/differentiation factor‐15, glial cell line‐derived neurotrophic factor, growth hormone 1, heparin‐binding EGF‐like growth factor, hepatocyte growth factor, insulin‐like growth factor‐binding protein, insulin growth factor‐1, macrophage colony‐stimulating factor 1 receptor, NGF receptor, neurotrophin‐3 (NT‐3), NT‐4, Platelet‐derived growth factor A chain, placenta growth factor, stem cell factor (SCF), SCF receptor, transforming growth factor alpha (TGF‐α), TGF beta‐1 (TGF‐β1), TGF beta‐3 (TGF‐β3), and VEGF.

### 2.3 Formulation of the cell‐laden collagen/β‐TCP and bone‐derived dECM/β‐TCP bioinks

As constituents of the control bioink, type‐I collagen derived from porcine skin (MS‐Bio, South Korea) and bioceramic (β‐TCP, Sigma‐Aldrich) were used. Collagen of 5 wt% and 5 wt% dECM solution, neutralized with 10× enriched Dulbecco's modified Eagle's medium (Sigma‐Aldrich), were physically mixed with various weight fractions (0–40 wt%) of β‐TCP. hDPSCs (ATCC, USA) at the density of 1 × 10^7^ cells/ml were mixed into the solutions [collagen (5 wt%)/β‐TCP and dECM (5 wt%)/β‐TCP].

### 2.4 Rheological properties of bioinks

A rotational rheometer (Bohlin Gemini HR Nano; Malvern Instruments) equipped with cone‐and‐plate geometry (cone angle of 4°, diameter of 40 mm, and gap of 150 μm) was used to measure the rheological properties of the prepared bioinks. The storage modulus (G') of the various bioinks was measured in terms of shear stress (0.1–1000 Pa, temperature: 25°C, frequency: 1 Hz) and temperature sweeps (10–50°C, frequency: 1 Hz, strain: 1%). The shear stress value at the limit of the linear viscoelastic region for the G' vs. shear stress curves is marked as the yield stress (*τ*_*y*_). All tests were performed in triplicate.

### 2.5 Fabrication of cell‐laden biocomposites

Cell‐laden structures (10 × 10 × 1.3 mm^3^) were fabricated using a three‐axis robot system (DTR3–2210 T‐SG; DASA Robot) equipped with a dispensing system (AD‐3000C, Ugin‐tech) and a 25G dispensing needle (inner diameter: 250 μm). The printing conditions, including pneumatic pressure and processing temperature, were appropriately selected, except for the nozzle moving speed of 10 mm/s. After fabricating the cell‐laden constructs, crosslinking was performed using 1 mM genipin solution (Challenge Bioproducts) in a medium for 30 min at 37°C with 5% CO_2_.

### 2.6 Characterization of cell‐laden composite scaffolds

To visualize the surface morphologies, optical microscope (BX FM‐32; Olympus) and field emission scanning electron microscopy (FESEM; JSM‐7500f; JEOL Ltd.) were used. In addition, the distribution of elemental phosphate (P) and calcium (Ca) was evaluated using energy‐dispersive spectroscopy (EDS).

To characterize the crystal peaks of β‐TCP, Wide‐angle X‐ray diffraction (X'Pert PRO MRD; PANalytical, UK) with CuKα radiation, under the beam conditions of 40 kV and 20 mA with spectrum collection at 2θ = 20–40° and the step size of 0.1° was used.

The compressive properties of the cell‐laden structures (6 × 6 × 4 mm) in a wet state were measured using a universal testing instrument (SurTA; Chemilab). Briefly, the compression speed was set at 0.5 mm/s and the compressive modulus was calculated using 5%–10% strain of the stress–strain curves.

### 2.7 In vitro cell culture

hDPSCs (Lonza) were cultured in Minimum Essential Medium alpha (MEM‐α; Gibco, Thermo Fisher Scientific) containing 10% fetal bovine serum (FBS) (Gemini Bio‐Products), and 1% penicillin/streptomycin (Thermo Fisher Scientific) was used as the cell growth medium (GM). Cells (passage 4) were used in the bioinks.

The cell‐laden constructs were cultured in a 6‐well culture plates supplemented with GM and incubated at 37 °C in 5% CO_2_. The medium was replaced every 2 days. To induce osteogenic differentiation of the hDPSCs, 100 μM dexamethasone (Sigma‐Aldrich), 10 mM β‐glycerophosphate (Sigma‐Aldrich), and 50 μM ascorbate‐2‐phosphate (Sigma‐Aldrich) were mixed with the GM. The fabricated cell‐laden structures were cultured in osteogenic differentiation medium (DM) after 7 days of culture. The DM was changed every 2 days.

### 2.8 In vitro cellular activities

Cell viability was assessed using a live (green)/dead (red) assay (LIVE/DEAD™ Viability/Cytotoxicity Kit, for mammalian cells, Thermo Fisher Scientific). Briefly, the hDPSCs laden in the biocomposites were incubated in 0.15 mM calcein AM and 2 mM ethidium homodimer‐1 at 37°C for 30 min and the stained cells were visualized using confocal microscopy (LSM 700; Carl Zeiss). To calculate cell viability, three samples were used to capture more than three images per specimen, while ImageJ software (National Institutes of Health) was used to count the number of live/dead cells.

To visualize the morphologies of the hDPSCs, the cell‐laden constructs were incubated with Diamidino‐2‐phenylinodole (DAPI, blue; Invitrogen) diluted in PBS with dilution ratio of 1:100 and Alexa Fluor 594 fluorescein phalloidin (red) (Invitrogen) diluted in PBS with dilution ratio of 1:100 to stain the nuclei (blue) and cytoskeleton (red), respectively. The morphology of the cells was visualized using a confocal microscope.

MTT assay (*n* = 4) (Cell Proliferation Kit I; Boehringer Mannheim) was used to observe cell proliferation. For the MTT assay, the cell‐laden structures (*n* = 4) were treated with a mixture of MEM‐α (180 μl) and MTT solution (20 μl) for 4 h in an incubator. A 200‐μl volume of the lysis buffer was then added to the reaction buffer. The optical density at 570 nm was measured using a microplate reader (EL800, BioTek).

### 2.9 Osteogenic activities

The alkaline phosphatase (ALP) activity was assessed via measuring the release of p‐nitrophenol (pNP) released from pNP phosphate (pNPP). Briefly, the cell‐laden constructs were rinsed using PBS, followed by incubation using Tris buffer (10 mM, pH 7.5) containing 0.1% Triton X‐100 for 10 min. Afterwards, 100 μl of the lysate was added into 96‐well culture plate containing equal volume of pNPP solution. Then, the ALP activity was quantified using aa microplate spectrophotometer using a wavelength of 405 nm. Furthermore, the cell‐laden biocomposite were stained with ALP by twice rinsing the composite using PBS. Then, ALP buffer (100 mM Tris‐Cl, pH 9.5, 100 mM NaCl, and 10 mM MgCl_2_) was used for equilibration. Then, the biocomposites were immersed in BCIP/NBT (Sigma‐Aldrich) for 30 min, followed by enzymatic activity termination via rinsing the samples using PBSS containing 20 mM EDTA. The ALP stained biocomposite were visualized using an optical microscope.

To evaluate the degree of calcium mineralization, the cell‐laden biocomposites were stained with Alizarin red S staining. Briefly, the biocomposites were rinsed twice using PBS, followed by fixation in 3.8% formaldehyde solution (Sigma‐Aldrich). Then the biocomposites were stained with 40 mM Alizarin red S with pH of 4.2 for 60 min. The stained biocomposites were visualized using an optical microscope. To quantify the degree of mineralization, the Alizarin red S stained biocomposites were rinsed three times with distilled water and destained using 10% cetylpyridinium chloride in 10 mM sodium phosphate buffer (pH 7.0) for 15 min. The degree of calcium mineralization was measured using a microplate spectrophotometer at wavelength of 562 nm. To assess the mineralization by the cells, the optical density value of the cell‐free composites was taken away from the value of the cell‐laden structures. All data values were defined as ± SD (*n* = 5).

### 2.10 Immunofluorescence

The hDPSCs on the biocomposites were stained with (*OPN*) and dentin sialophosphoprotein (*DSPP*) immunofluorescence to assess degree of osteogenesis and odontogenesis. Briefly, the biocomposites were rinsed twice using PBS and hDPSCs were fixated using 3.7 formaldehyde for 60 min. Then, the biocomposites were permeabilized using 2% Triton X‐100 for 1 h and treated using 2% bovine serum albumin (Sigma‐Aldrich) for 1 h. Subsequently, the biocomposites were treated with anti‐OPN and anti‐DSPP primary antibodies (5 mg/ml; Abcam) overnight at 4°C. Afterwards, the biocomposites were rinsed twice using PBS then treated with Alexa Fluor 488 conjugated‐mouse antibodies (dilution ratio of 1:50 in PBS) and Alex Fluor 594‐rabbit antibodies (dilution ratio of 1:50 in PBS) for 1 h for OPN and DSPP immunofluorescent staining respectively. Then the samples were counterstained with 5 mM DAPI. The images were visualized using confocal microscope and ImageJ software was used to evaluate the OPN‐ and DSPP‐positive areas.

### 2.11 Real‐time polymerase chain reaction (RT‐PCR)

To evaluate the various osteogenic and odontogenic markers, including osteopontin (*OPN*), osteocalcin (*OCN*), biglycan (*BGN*), dentin sialophosphoprotein (*DSPP*), and dentin matrix acidic phosphoprotein‐1 (*DMP‐1*), RT‐PCR (Applied Biosystems) was performed after 21 and 28 days of culture. Briefly, the total RNA was isolated from the cultured hDPSCs on the biocomposites using TRI reagent (Sigma‐Aldrich), followed by purity and concentration evaluation using spectrophotometry (FLX800T; Biotek). Then, reverse transcription system using ReverTra Ace qPCR RT Master Mix (Toyobo, Japan) was utilized to synthesize complementary DNA (cDNA) from the RNase‐free DNase‐treated total RNA. Finally, StepOnePlus Real‐Time PCR System (THUNDERBIRD® SYBER® qPCR Mix (Toyobo, Japan) was used to quantify the gene expressions. The gene‐specific primers sequences are listed in Table 1.

**TABLE 1:** Primers sequences used for hDPSCs RT‐PCR

Gene	Host	Primer sequence	
*ACTB*	Human	Forward	5′‐tcc aaa tat gag atg cgt tgt t‐3′
		Reverse	5′‐tgc tat cac ctc ccc tgt gt‐3′
*ALP*	Human	Forward	5′‐ggc acc tgc ctt act aac tcc‐3′
		Reverse	5′‐ctta gcc acg ttg gtg ttg a‐3′
*BMP‐2*	Human	Forward	5′‐gac tgc ggt ctc cta aag gtc‐3′
		Reverse	5′‐gga agc agc aac gct aga ag‐3’
*OCN*	Human	Forward	5′‐ tga gag ccc tca cac tcc tc‐3’
		Reverse	5′‐ acc ttt gct gga ctc tgc ac‐3’
*OPN*	Human	Forward	5′‐aag ttt cgc aga cct gac atc‐3’
		Reverse	5′‐ggg ctg tcc caa tca gaa gg‐3’
*DSPP*	Human	Forward	5′‐gac aca tgc tgt tgg gaa ga‐3’
		Reverse	5′‐ctc ttt acc ttc gtt gcc ttt c‐3’
*DMP‐1*	Human	Forward	5′‐ttc ttt gtg aac tac gga ggg ta‐3’
		Reverse	5′‐cag gat aat ccc caa agg aac‐ 3’
*BGN*	Human	Forward	5′‐ctc cca gac ctc aag ctc ct −3’
		Reverse	5′‐tgg gac aga agt cgt tga ca‐3’

*ACTB,* Beta actin*; ALP*, Alkaline phosphatase; *BMP‐2*, Bone morphogenetic protein 2; *OCN*, Osteocalcin; *OPN*, Osteopontin; *DSPP*, Detin sialophosphoprotein; *DMP‐1*, Dentin matrix acidic phosphoprotein 1*; BGN*, Biglycan.

### 2.12 In vivo implantation of the hDPSCs‐laden composite structure

The bioprinted collagen/β‐TCP and dECM/β‐TCP composite structures (8 × 8 × 4 mm^3^) with hDPSCs were implanted in dorsal subcutaneous pockets of athymic nude mice (Orient Science Co.). All experimental animals used in this study were cared for under a protocol approved by the Institutional Animal Care and Use Committee of Chonbuk National University Hospital (JBUH‐IACUC‐2020‐10‐1). A total of 12 animals (*n* = 4 per group) were used in the in vivo evaluation: three groups – (i) dECM without cells, (ii) CTS‐20, and (iii) dECM‐20. The implanted composite structures were harvested after 8 weeks post implantation.

### 2.13 Histology and immunohistochemistry examination

The harvested samples were fixed in 10% neutral buffered formalin (NBF, Leica Biosystem) at room temperature for 24 h. Afterward, decalcified in decalcifying solution (Sigma‐Aldrich) at room temperature for 7 days. The samples were paraffin‐embedded and sectioned into 5 μm thick sections. Deparaffinized sections were stained with Hematoxylin and Eosin (H&E) staining.

For immunohistochemistry, deparaffinized sections were rehydrated and subjected to antigen retrieval at 37°C for 30 min. The samples were protein blocked with a serum‐free blocking solution at room temperature for 1 h, then, incubated with anti‐OPN (1:100 dilution, Abcam, MA, USA), anti‐OCN (1:50 dilution, Santa Cruz, CA, USA), anti‐DSPP (1:50 dilution, Santa Cruz, CA, USA), and ani‐DMP‐1 (1:500 dilution, Santa Cruz, CA, USA) primary antibodies at 4°C overnight. The samples were incubated with the secondary biotinylated anti‐goat antibody (BA‐5000, Vector Laboratories) for 30 min. Streptavidin‐conjugated horseradish peroxidase (SA‐5704, Vector Laboratories) was added for 30 min, and the samples were stained with 3,3′‐Diaminobenzidine (DAB). Then, Gill's hematoxylin was used to counterstain the cell nuclei. The stained sections were visualized under the light microscope and the positive areas of OPN, OCN, DSPP, and DMP‐1 were measured using Image J software.

### 2.14 Statistical analyses

SPSS software (SPSS, Inc.) was used to conduct statistical analyses. A single factor analysis of variance was employed, and a value of p* < 0.05, was considered statistically significant.

## 3 RESULTS AND DISCUSSION

### 3.1 Appropriate concentration of bioceramic in 3D‐printed scaffold laden with hDPSCs

In a previous study, we demonstrated an optimal printing method using bioinks (collagen/hASC/β‐TCP) for obtaining multilayered cell‐laden scaffolds.^25^ Under selected printing parameters, we were able to determine the appropriate weight fraction of β‐TCP in the bioinks considering the safe in‐situ cell viability (over 90%) after printing and stable 3D structural formation. Therefore, we need to establish the most suitable β‐TCP concentration in a bioink containing hDPSCs for dentin regeneration, because cells in the bioink can respond differently to bioceramic concentration laden in a bioink as well as the applied printing conditions.

**FIGURE 1:**
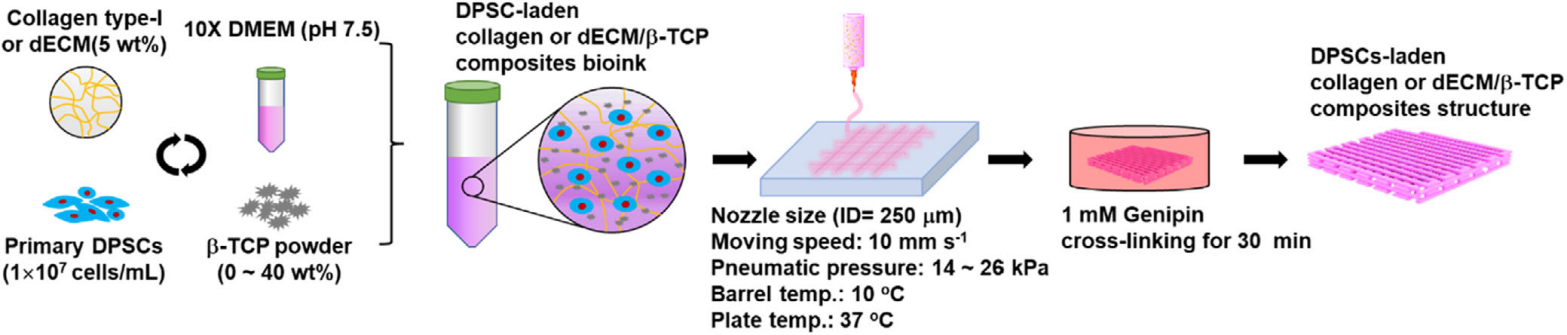
Schematic of bioink formulation using collagen type‐I, bioceramic, and human dental pulp stem cells (hDPSCs) and the procedure (printing and crosslinking) for fabricating cell‐laden biocomposite

To observe the effect of the ceramic concentration on the hDPSC activities and 3D mesh structural formation, bioinks containing 5 wt% collagen/hDPSCs (1 × 10^7^ cells/ml) and various weight fractions (0–40 wt%) of β‐TCP were used, as shown in Figure 1.

**FIGURE 2:**
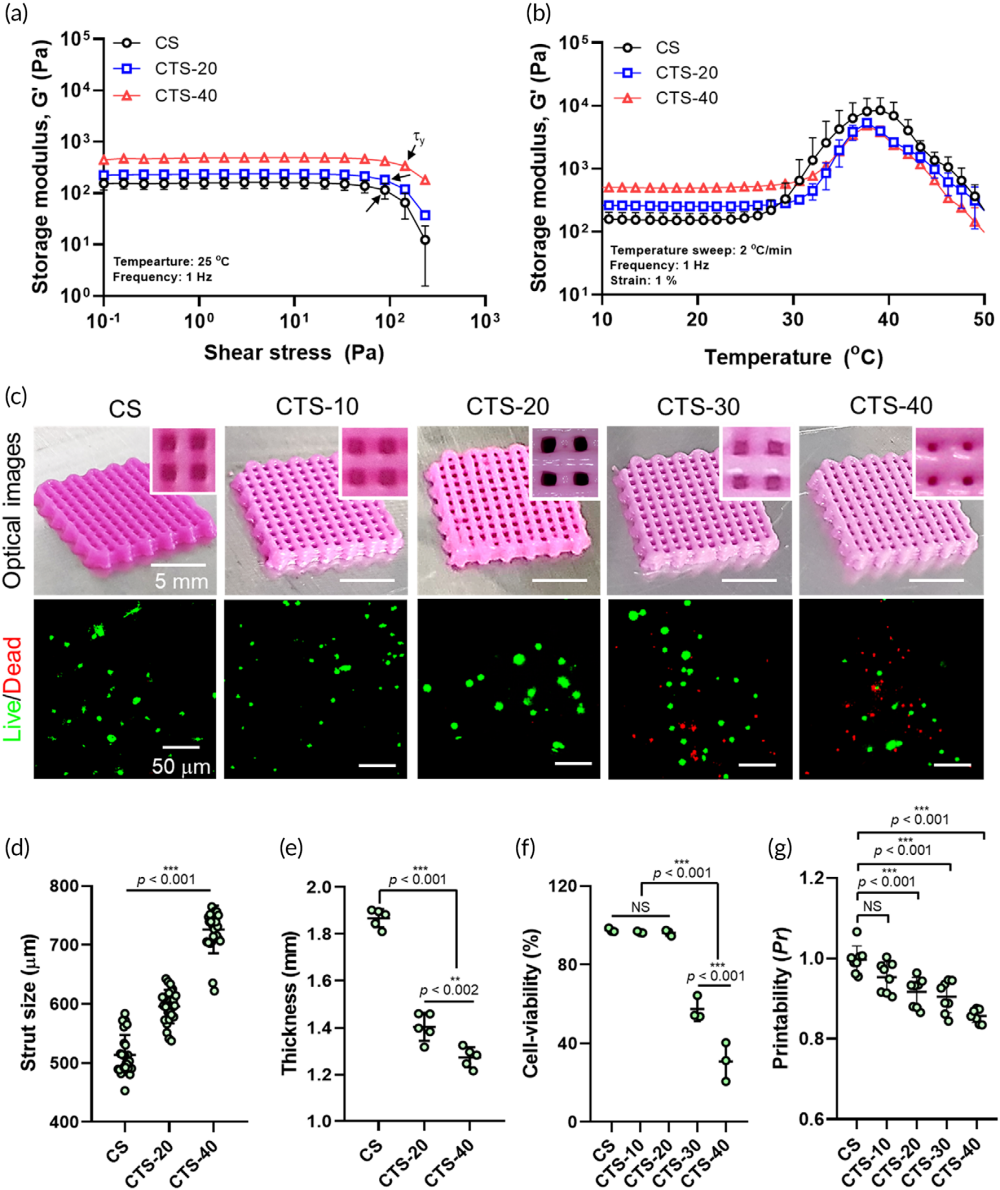
Storage modulus (G′) of bioinks (collagen/β‐TCP/hDPSCs) for various weight fractions of the β‐TCP (CS: 0 wt%, CTS‐20: 20 wt%, and CTS‐40: 40 wt%) with (a) shear stress and (b) temperature sweeps. (c) Optical and live (green)/dead (red) of the printed biocomposites for various weight fractions of β‐TCP. Geometrical sizes (d) strut size, (e) thickness), (f) cell viability, and (g) printability of the printed biocomposites were measured for various bioceramic concentrations

Figure 2a‐b show the rheological properties of the hDPSC‐laden bioinks (CS: pure collagen/hDPSCs, CTS‐20: collagen/β‐TCP‐20 wt%/hDPSCs, CTS‐40: collagen/β‐TCP‐40 wt%/hDPSCs) with various β‐TCP concentrations (0, 20, and 40 wt%) for shear stress and temperature sweeps, respectively. As shown in Figure 2a, the bioink with a higher concentration of bioceramic showed a significantly higher storage modulus (G') at 25°C, whereas a similar yield stress (*τ*_*y*_) was observed. For the temperature sweep result, G' peaks of the bioinks were observed near 38 °C owing to collagen in the bioink.^19^, ^26^ Gelation was the highest in the pure collagen bioink, and as the ceramic concentration increased, the modulus was significantly reduced owing to the thermal conduction of the embedded ceramics in the bioink (Figure 2b). Based on the rheological properties, the printing temperature in the barrel and printing stage were set to 10 and 38 °C, respectively, to avoid high shear stresses in the nozzle and to ensure mechanical sustainability of the printed structure in the working plate.

In biomedical scaffolds, pore geometries, including pore size and strut size, are important design parameters. To circumvent the effect of pore geometry on the cellular responses of the laden cells, we set the pore (500 μm) and strut (500 μm) sizes by controlling the printing parameters, as shown in Table 2. Figure 2c shows the optical and live (green)/dead (red) images of the 3D‐printed biocomposite structures for various bioink formations. After the crosslinking process was complete, the structures exhibited slightly different pore geometries than the initially printed structure, because they were gradually squeezed owing to the weight of the embedded ceramic fraction. This squeezing was more accelerated in the structures with relatively higher bioceramic concentrations than in the pure collagen bioink (Figure S1a,b). According to this result, the strut size and thickness of the printed composites differed slightly from those of the originally designed geometry (Figure 2d‐e). In addition, the initial cell viability is an important criterion for determining printing stability. Figure 2f shows the initial cell viability, determined using the live/dead images of the hDPSCs in Figure 2c, after the crosslinking procedure. As indicated by the results, the cell viability at higher ceramic concentrations (bioceramic >30 wt%) was significantly reduced due to the higher wall shear stresses in the nozzle, signifying that the concentration of the bioceramic (below 20 wt%) was safe for the laden cells. Furthermore, as expected from the strut size and thickness of the printed composite, the printability (Pr) (Pr values of <1, =1, and >1 indicate that the bioink has a low viscosity, an appropriate viscosity, and a high viscosity, respectively)^27^ of the biocomposite gradually decreased with increasing ceramic concentration (Figure 2g). Considering the 3D structural shape‐ability and hDPSC viability laden in the printed structure, we used the bioceramic concentration of 20 wt% in the hDPSC‐laden bioink for the following analyses.

**TABLE 2:** Printing conditions of the CS, CTS‐10, CTS‐20, CTS‐30, and CTS‐40 composite structures

	CS	CTS‐10	CTS‐20	CTS‐30	CTS‐40
Concentration of β‐TCP (wt%)	‐	10	20	30	40
Nozzle diameter (mm)	0.25	0.25	0.25	0.25	0.25
Pneumatic pressure (kPa)	14	15	17	24	26
Nozzle moving speed (mm/s)	10	10	10	10	10
Working plate temperature (°C)	38	38	38	38	38
Barrel temperature (°C)	10	10	10	10	10

### 3.2 Effect of β‐TCP on hDPSC cellular activity in 3D‐printed cell‐laden structure

**FIGURE 3:**
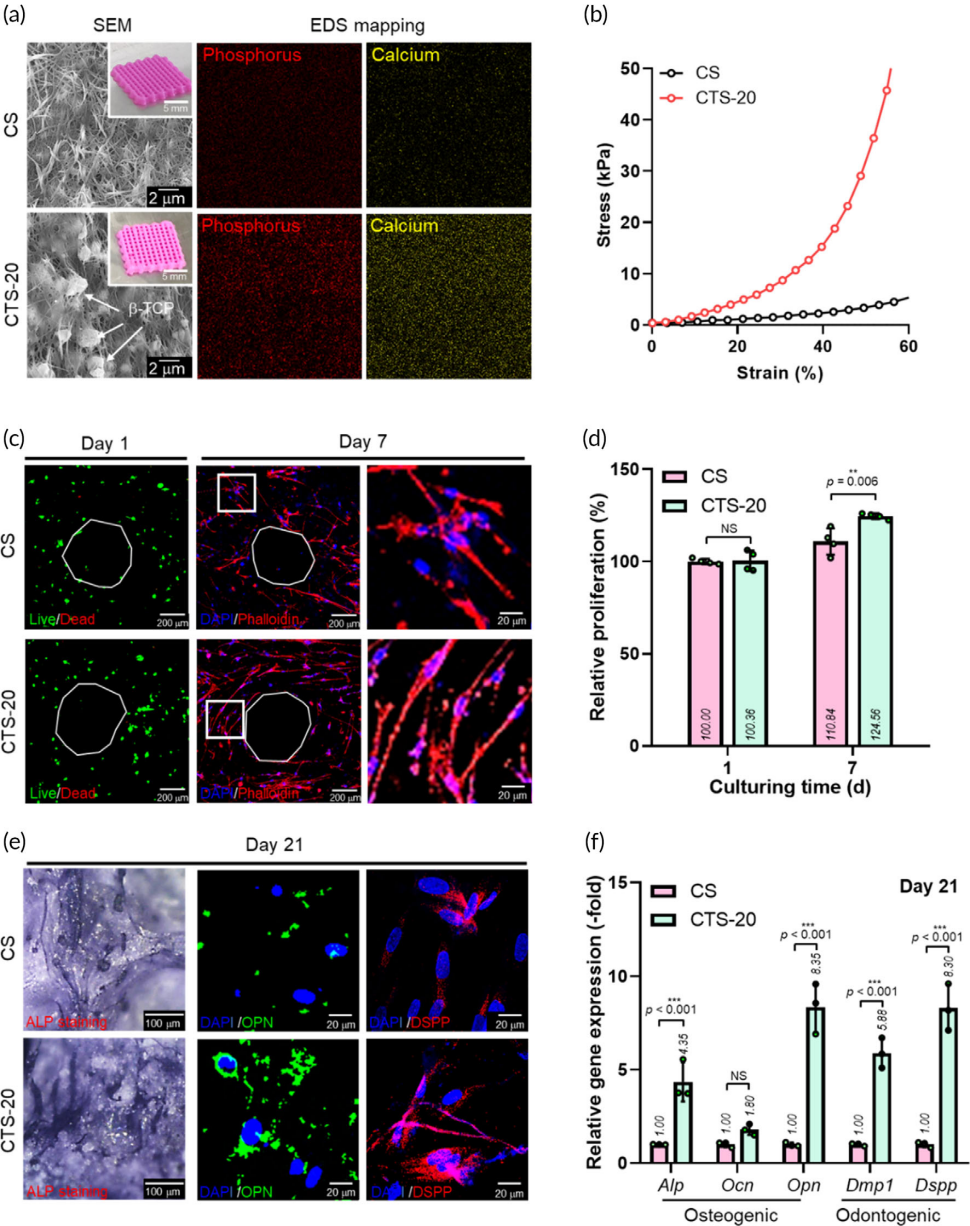
(a) SEM/optical images and EDS mapping (phosphorus and calcium) of CS (pure cell‐laden collagen) and CTS‐20. (b) Compressive stress–strain curves for CS and CTS‐20. (c) Live/dead images at 1 day and DAPI (blue)/phalloidin (red) images at 7 days and (d) relative proliferation, determined by MTT assay, for CS and CTS‐20. (e) Alkaline phosphatase (ALP), osteopontin (OPN), and dentin sialophosphoprotein (DSPP) staining images at 21 days and (f) osteo/odontogenic gene expression at 21 days for the CS and CTS‐20 scaffolds

To evaluate the effect of β‐TCP on the hDPSC cellular responses in the 3D‐printed scaffolds, hDPSC‐laden mesh scaffolds with and without β‐TCP (20 wt%) scaffolds were fabricated, and their 3D optical/*SEM* images and EDS results for the scaffold surface are shown in Figure 3a. In the *SEM* images, both the CS and CTS‐20 structures display fibrillated collagen components; however, for the CTS‐20 biocomposite, a mixed structure of fibrillated collagen and embedded ceramic particles can be observed. Furthermore, the EDS results showing the distribution of P and Ca were assessed in the CS and CTS‐20 structures. From the results for the CTS‐20 scaffold, homogeneously distributed P and Ca were observed on the surface of the CTS‐20 scaffold, while the elements were not detected in the CS scaffold.

Figure 3b shows the stress–strain curves in compression mode for wetted CS and CTS‐20 scaffolds, with the compressive speed of 0.5 mm/s. The compressive modulus values of CS and CTS‐20 were 5.6 ± 0.8 and 27.9 ± 2.2 kPa, respectively. The increased modulus of CTS‐20 was due to the embedded bioceramics in the bioink. We can expect that this mechanical improvement not only supports the handling of the scaffold but also affects the cellular activities of hDPSCs via mechano‐transduction signaling.^28^

Figure 3c shows the fluorescence images of live (green)/dead (red) and DAPI (blue)/phalloidin (green) for the CS and CTS‐20 scaffolds. As shown in the live/dead images at 1 d, the bioprinted hDPSCs were sufficiently safe in both structures: CS (cell viability = 96.4% ± 1.8%) and CTS (95.8% ± 0.3%). Furthermore, the nucleus/F‐actin images at 7 days indicated that the cells proliferated well in both structures. However, the morphology of the cytoskeleton in the CTS‐20 structure was more stretched and developed uniaxially than the CS structure. This phenomenon is likely due to the higher mechanical stiffness of the CTS‐20 structure than that of the CS structure. Figure 3d shows the relative cell proliferation, determined via MTT assay, and the value was normalized to the optical density of CS at 1 day. As shown in the results, cell proliferation at 7 days was significantly higher in the CTS‐20 than in the CS. We believe that the addition of β‐TCP enhanced the mechanical stiffness of collagen hydrogel and improved the in vitro cellular activities, including cell morphology and cell growth.^29^ Similar results have also been reported for spongy‐shaped collagen/β‐TCP composites, where the mechanical stiffness of the composite scaffold induced significantly higher cell growth than a pure collagen scaffold with relatively low mechanical stiffness.^30^

Figure 3e shows the immunofluorescence staining for observation of the ALP, OPN, and DSPP expressions at 21 days in the CS and CST‐20 scaffolds. As shown in the images, more positive staining for ALP, OPN, and DSPP were detected in the CTS‐20 construct, as compared with those for CS. These results indicate that higher osteogenic/odontogenic differentiation of the cells laden in the CTS‐20 structures was possible. In addition, we measured the osteogenic (*ALP*, *OCN*, and *OPN*) and odontogenic (*DMP‐1* and *DSPP*) gene expressions using RT‐PCR (Figure 3f). As expected, the immunofluorescence images indicate that the CST‐20 structure featured significantly higher osteogenic/odontogenic gene expressions than the CS scaffold.

We believe that the cellular responses of hDPSCs can be directly affected by the osteoinductive and osteoconductive β‐TCP components embedded in the bioink.

### 3.3 Strategy to induce higher cellular activities using bone‐derived dECM in hDPSC‐laden structures

In general, dECM biomaterials have been widely used in tissue engineering because they possess outstanding biochemical and physiological components derived from native tissues^31^ For regenerative dentistry, dentin‐derived dECM (d‐dECM) has been used as a potential bioink mixed with a 1:1 ratio [alginate hydrogel (3% w/v) and d‐dECM] to regenerate dentin.^21^ The cell‐laden scaffold using the d‐dECM‐based bioink showed good cytocompatibility and odontogenic activity.^21^ However, this approach of using the dentin‐derived dECM‐based bioink can be highly inefficient because the yield rate for obtaining dentin‐derived dECM from native dentin tissue is considerably lower compared to that of bone‐derived dECM (Figure S2).

**FIGURE 4:**
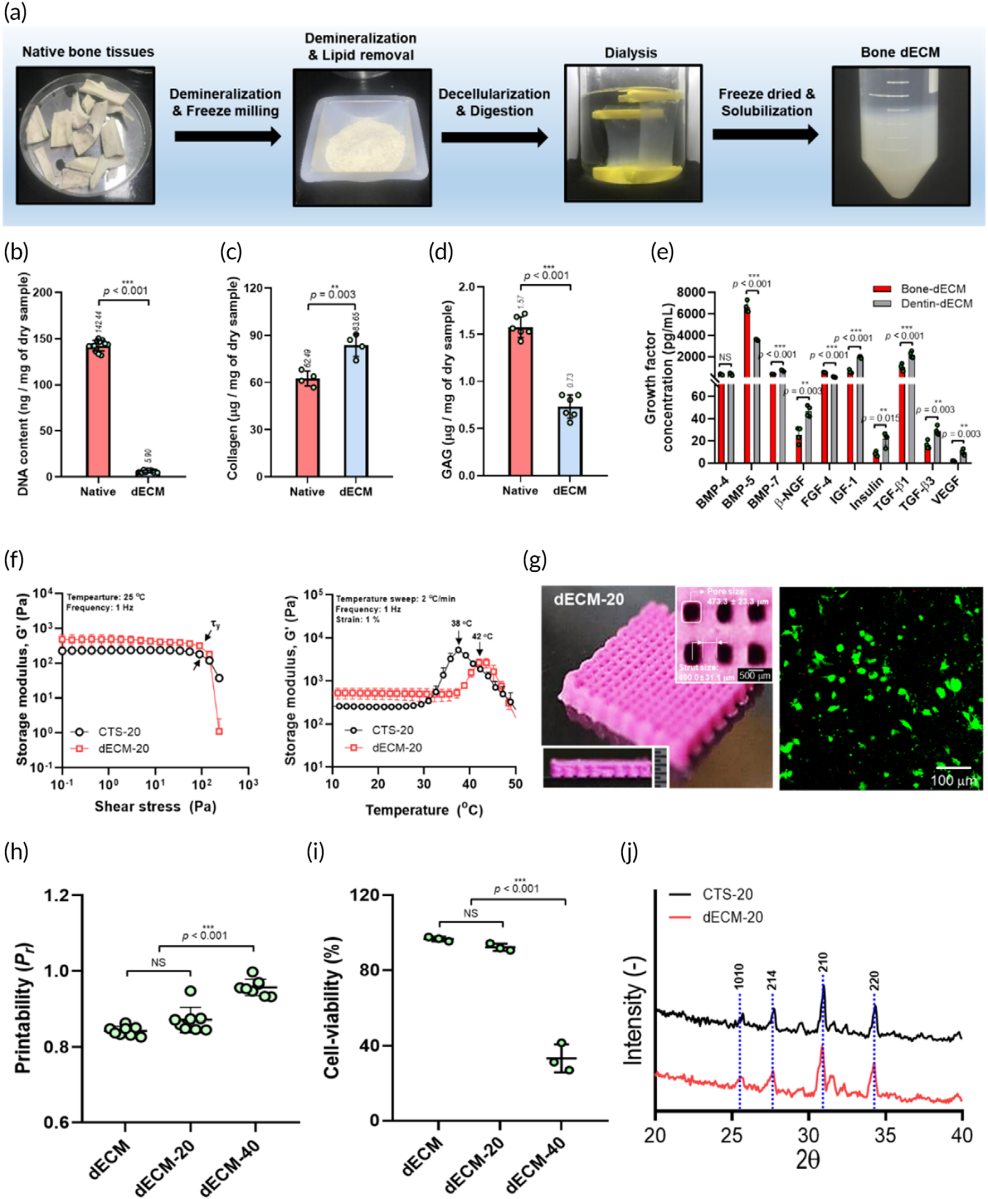
(a) Schematic of the decellularization process using bovine‐bone tissue and (b) DNA, (c) collagen, and (d) glycosaminoglycan (GAG) contents for native tissue and decellularized extracellular matrix (dECM). (e) Comparison of growth factor concentration for porcine‐derived dECM (bone‐dECM) and dentin‐derived dECM (dentin‐dECM). (f) Comparison of storage modulus values of CTS‐20 and dECM‐20 for shear stress and temperature sweeps. (g) Optical and live/dead images for dECM‐20 biocomposite. (h) Printability and (i) cell viability for dECM‐based biocomposites with various weight fractions of β‐TCP. (j) X‐ray diffraction results of CTS‐20 and dECM‐20

To overcome this low yield rate, we used dECM derived from bovine bone tissues. To successfully obtain dECM biomaterials, complete elimination of the cellular components is required, without a significant loss of the bioactive components such as collagen, fibronectin, elastin, GAG, and proteoglycans. The images in Figure 4a show the decellularization procedure from native porcine bone tissue, and Figure 4b‐D show the relative DNA contents, collagen, and GAGs, respectively, of the native bone tissue and decellularized bone. If the cellular component has a dry weight of less than 50 ng/mg, the decellularization process is expected to be satisfactory.^32^ As shown in Figure 4b, the DNA content had a dry weight of approximately 5.9 ± 1.2 ng/mg, and the collagen and GAGs for the native and dECM were well retained in the dECM. In addition, to compare the growth factors of the extracted bone‐derived dECM and dentin‐derived dECM (d‐dECM) biomaterials, we performed decellularization of the dentin tissue (Figure S3a), and the remaining components are shown in Figure S3b‐d. After the decellularization process, various growth factors of d‐dECM and dECM were assessed (Table 3). The growth factors for d‐dECM were similar to those of the bovine‐bone‐derived dECM. Although the known odontogenic growth factors, bFGF and EGF,^4^ were not observed in the bone‐derived dECM, other key growth factors, including BMPs, used as a suitable cellular niche to modulate osteo/odontogenic differentiation, were similar for both dECMs (Figure 4e). According to Lee et al.,^5^ BMP was used to fabricate a hDPSC‐laden methacrylated‐gelatin (GelMa) structure, and the BMP peptide induced significant osteo/odontogenic differentiation of hDPSCs, as compared with the pure cell‐laden GelMa structure. Similarly, hDPSCs cultured on the surface of bone‐derived dECM spongy scaffolds significantly upregulated the expressions of odontogenic genes, DSPP, and DMP‐1.^4^ Therefore, we used bone‐derived dECM to modulate hDPSCs into osteogenic and odontogenic differentiation as a potential bioink.

**TABLE 3:** Growth factors of bone‐derived dECM and dentin‐derived dECM

[pg mL^−1^]	Bone‐dECM [*n* = 4]	Dentin‐dECM [*n* = 4]	LOD
Amphiregulin	7.3 ± 2.4	25.3 ± 6.5	24.7
BDNF	2.6 ± 0.1	3.8 ± 0.4	4.4
bFGF	0.0 ± 0.0	3.3 ± 2.7	9.7
BMP‐4	357.7 ± 61.4	399.4 ± 94.3	975.9
BMP‐5	6611.4 ± 508.2	3587.4 ± 62.7	2277.2
BMP‐7	371.6 ± 16.2	708.3 ± 62.1	397.8
β‐NGF	25.5 ± 7.0	46.8 ± 4.9	91.9
EGF	0.0 ± 0.0	0.2 ± 0.1	0.4
EGF receptor	4.0 ± 0.2	41.5 ± 3.3	45.2
EG‐VEGF	7.0 ± 2.8	11.3 ± 1.6	15.8
FGF‐4	541.9 ± 32.9	113.2 ± 31.9	274.0
FGF‐7	35.7 ± 12.8	106.6 ± 25.8	119.3
GDF‐15	2.3 ± 0.5	4.8 ± 1.2	19.0
GDNF	0.0 ± 0.0	7.6 ± 1.1	22.1
GH1	52.8 ± 8.8	57.7 ± 3.3	88.7
HB‐EGF	3.1 ± 1.8	9.0 ± 2.8	27.1
HGF	9.4 ± 3.0	15.9 ± 2.4	18.0
IGFBP‐1	9.7 ± 2.6	15.5 ± 3.7	17.6
IGFBP‐2	197.0 ± 17.0	294.8 ± 63.2	186.3
IGFBP‐3	2984.4 ± 265.6	3899.5 ± 947.6	5406.9
IGFBP‐4	1348.5 ± 390.0	2185.9 ± 191.9	1761.6
IGFBP‐6	454.6 ± 73.7	1012.7 ± 191.9	973.8
IGF‐1	605.8 ± 148.2	1992.3 ± 85.8	512.8
Insulin	8.4 ± 1.9	22.0 ± 6.00	62.6
MCSF R	55.2 ± 10.2	106.3 ± 26.7	187.2
NGF R	16.0 ± 2.5	18.1 ± 4.02	54.2
NT‐3	7.1 ± 1.5	29.2 ± 5.0	111.1
NT‐4	37.4 ± 7.8	66.1 ± 13.1	68.0
Osteoprotegerin	9.5 ± 1.6	13.1 ± 3.8	30.9
PDGF‐AA	1.0 ± 0.1	9.3 ± 2.9	72.3
PIGF	3.3 ± 0.9	5.3 ± 0.9	11.0
SCF	12.3 ± 2.1	19.4 ± 2.3	34.3
SCF R	3.4 ± 0.5	22.4 ± 8.00	79.6
TGFα	9.8 ± 1.00	34.0 ± 5.1	40.3
TGF β1	1025.1 ± 302.1	2194.4 ± 297.6	1207.8
TGF β3	16.3 ± 3.6	29.1 ± 3.8	36.6
VEGF	1.0 ± 0.4	9.9 ± 2.4	26.6
VEGF receptor 2	51.1 ± 7.7	65.0 ± 12.4	71.1
VEGF receptor 3	819.1 ± 146.3	1125.0 ± 105.1	732.0
VEGF‐D	42.9 ± 3.4	92.4 ± 8.8	83.5

Abbreviations: BDNF, Brain‐derived neurotrophic factor; bFGF, Basic fibroblast growth factor; BMP, Bone morphogenetic protein; EGF, Epidermal growth factor; EG‐VEGF, Endocrine gland‐derived vascular endothelial growth factor; FGF, Fibroblast growth factor; GDF‐15, Growth/differentiation factor 15; GDNF, Glial cell line‐derived neurotrophic factor; GH1, Growth hormone 1; HB‐EGF, Heparin‐binding epidermal growth factor‐like growth factor; HGF, Hepatocyte growth factor; IGF‐1, Insulin growth factor 1; IGFBP, Insulin‐like growth factor‐binding protein; LOD, limit of detection; MCSF R, Macrophage colony‐stimulating factor 1 receptor; NGF R, Nerve growth factor receptor; NT‐3, Neurotrophin‐3; NT‐4, Neurotrophin‐4; PDGF‐AA, Platelet‐derived growth factor A chain; PIGF, Placenta growth factor; SCF R, Stem cell factor receptor; SCF, Stem cell factor; TGF α, Transforming growth factor alpha; TGF β1, Transforming growth factor beta‐1; TGF β3, Transforming growth factor beta‐3; VEGF, Vascular endothelial growth factor; β‐NGF, Beta‐nerve growth factor.

The bone‐derived dECM was used as one of the new bioink components (dECM‐20: 5 wt% of dECM, 20 wt% of β‐TCP, and hDPSCs [1 × 10^7^ cells/ml]) to induce a high degree of cellular activity, cell growth, and differentiation. In Figure 4f, the rheological behavior of dECM‐20 (β‐TCP: 20 wt%) was compared with that of the CTS‐20 bioink using shear stress and temperature modes. By considering the rheological properties of the dECM‐based bioink, we selected the printing conditions shown in Table 4, and the dECM‐20 structure was stably fabricated with pores (473.3 ± 23.3 μm) and struts (490.0 ± 31.1 μm) (Figure 4g), and the laden cells in the biocomposite were alive (cell viability: > 95%). In addition, printability and cell viability for the dECM‐bioinks were significantly affected by the bioceramic weight fraction (Figure S4a‐c and Figure 4h‐i). However, unlike the CTS‐bioinks, the printability of the dECM‐bioink improved with increasing ceramic concentration. In particular, the low printability of the pure dECM‐bioink was observed to have a relatively lower modulus at the gelation temperature compared to that of CS‐bioink, because the collagen concentration in dECM was lower than that in the CS‐bioink (Figure S5). As shown in Figure 4j, X‐ray diffraction peaks for the fabricated biocomposites of CTS‐20 and dECM‐20 were measured to verify the crystal pattern of the embedded bioceramic component. Both biocomposites exhibited a typical pattern of β‐TCP, showing an orthorhombic crystal structure.

**TABLE 4:** Printing conditions of the CTS‐20 and dECM‐20 composite structures

	CTS‐20	dECM‐20
Concentration of β‐TCP (wt%)	20	20
Nozzle diameter (mm)	0.25	0.25
Pneumatic pressure (kPa)	17	22
Nozzle moving speed (mm/s)	10	10
Working plate temperature (°C)	38	42
Barrel temperature (°C)	10	10

**FIGURE 5:**
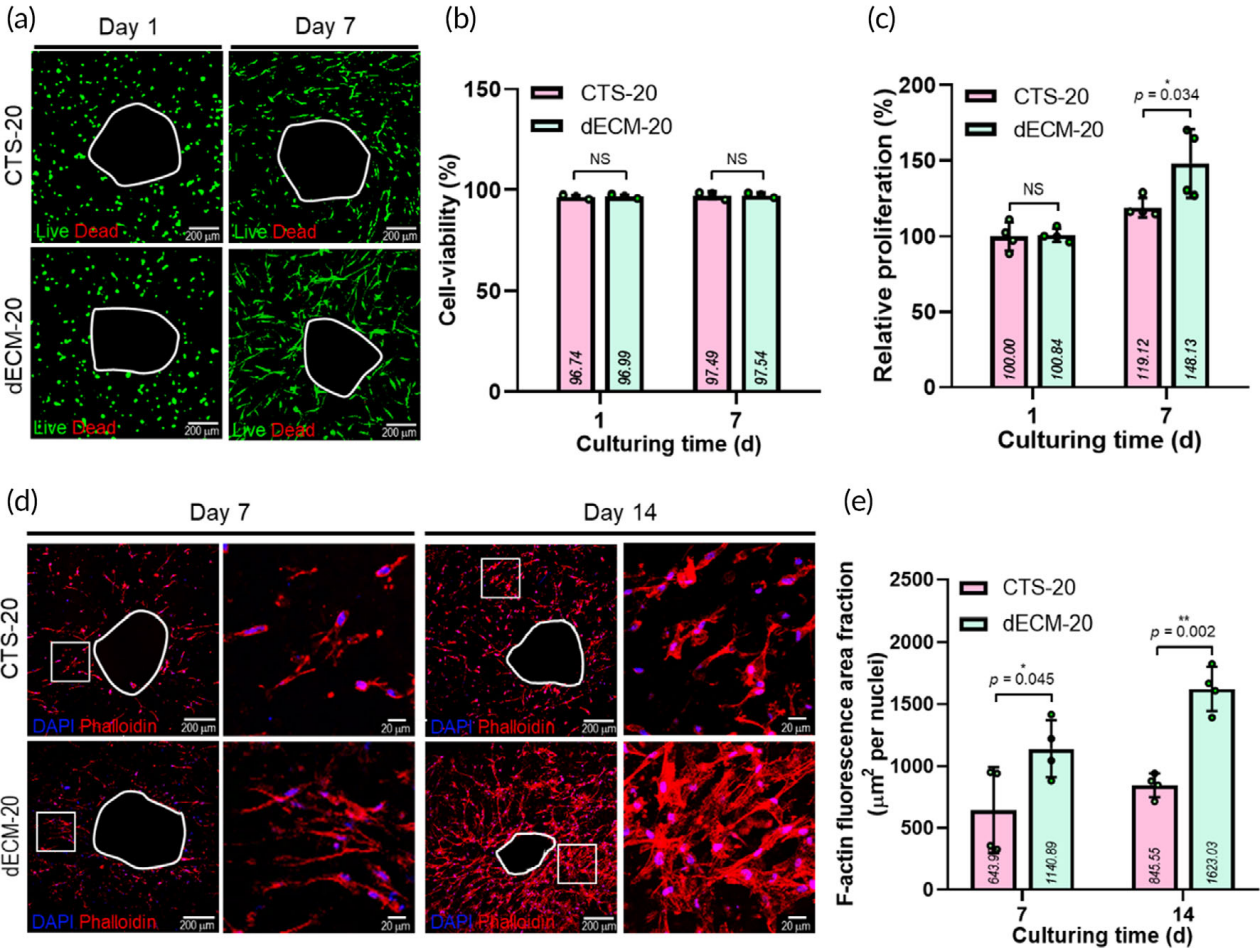
(a) Live/dead images, (b) cell viability, and (c) relative cell proliferation, determined by MTT assay, at 1 and 7 days for CTS‐20 and dECM‐20 biocomposites. (d) DAPI/phalloidin images and (e) F‐Actin area‐fractions at 7 and 14 days of CTS‐20 and dECM‐20 biocomposites

To observe the effect of the dECM component as a bioink on in vitro cellular activities, we used the collagen‐based biocomposite CTS‐20 (5 wt% of collagen/20 wt% of β‐TCP/hDPSCs) as a control. As shown in Figure 5a, the live/dead assay of the hDPSCs in the printed CTS‐20 and dECM‐20 structures on days 1 and 7 was performed. In both structures, the cells were mostly alive, and the cell viability measured from the live/dead images was approximately 96.7% ± 1.0% (CTS‐20) and 97.0% ± 0.9% (dECM‐20) after 1 day (Figure 5b).

Through Figure 5C, the proliferation of viable cells in the fabricated structure was assessed using the MTT assay. Both biocomposites showed an increasing trend of cell proliferation, but the rate of cell proliferation in dECM‐20 was significantly higher than that in CTS‐20, indicating that the proliferation ability of the hDPSCs was more affected in the dECM‐20 structure due to the outstanding biochemical cues of the dECM component. In addition, cell morphological shape and ECM generation were evaluated with the DAPI/phalloidin staining results at 7 and 14 days (Figure 5d). As expected, the cytoskeleton structure was more effectively spread and developed in the dECM‐20 structure than in CTS‐20 (Figure 5e).

### 3.4 Osteogenic and odontogenic differentiation of biocomposites

**FIGURE 6:**
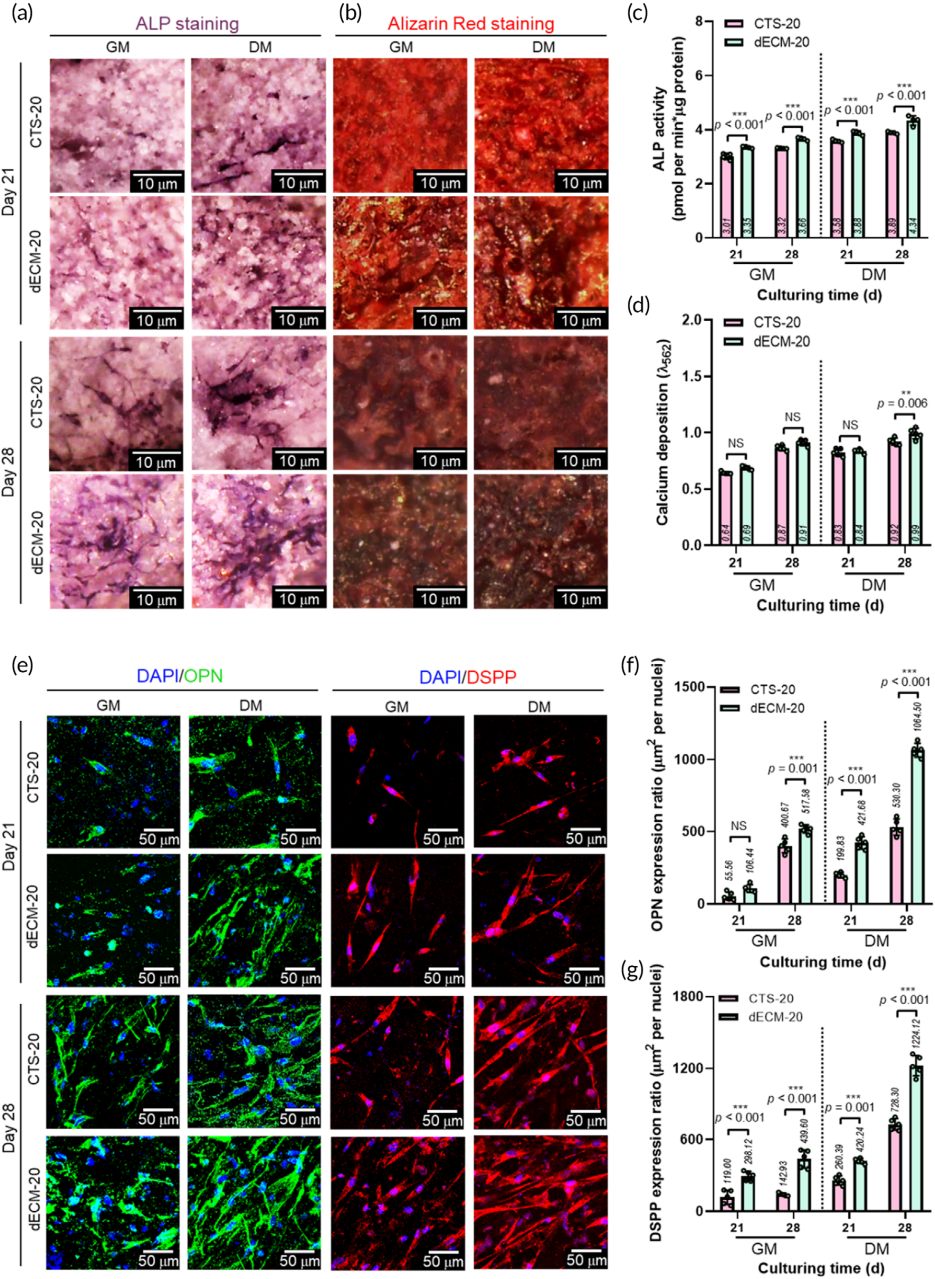
(a) ALP staining and (b) alizarin red staining at 21 and 28 days of the CTS‐20 and dECM‐20 biocomposites under different media (GM: growth medium and DM: differentiation medium). (c,d) Quantification of ALP and calcium deposition of the biocomposites (*n* = 5). (e) DAPI/OPN (green) and DAPI/DSPP (red) at 21 and 28 days of the CTS‐20 and dECM‐20 biocomposites under different media. (f,g) Quantification of OPN and DSPP (*n* = 5)

To observe the degree of osteogenic differentiation of the biocomposites, we conducted ALP and alizarin red S staining at 21 and 28 days (Figure 6a‐b). The culture medium was divided into two groups: GM and DM. As shown in the images, ALP activity and calcium deposition were observed in both structures, independent of the culture medium. However, the degree of ALP and calcium deposition were significantly higher in the dECM‐20 groups than in the CTS‐20 groups, and both structures in the DM exhibited significantly higher matrix formation of calcified tissues (Figure 6c‐d).

Osteo/odontogenic immunofluorescent staining (DAPI/OPN [green] and DAPI/DSPP [red]) of the structures was investigated at 21 and 28 days (Figure 6e). OPN and DSPP were quantified in the culture periods by assessing the green and red regions (Figure 6f‐g, respectively). In accordance with the previous results of ALP and calcium deposition, more apparently positive staining of OPN and DSPP was observed in dECM‐20 with increasing culture period from 21 to 28 days and when using DM. The results indicate that the osteogenic and odontogenic differentiation of hDPSCs could be clearly encouraged by the synergistic effect of bone‐derived dECM and β‐TCP components, and this trend was accelerated in the osteogenic differentiation culture medium.

**FIGURE 7:**
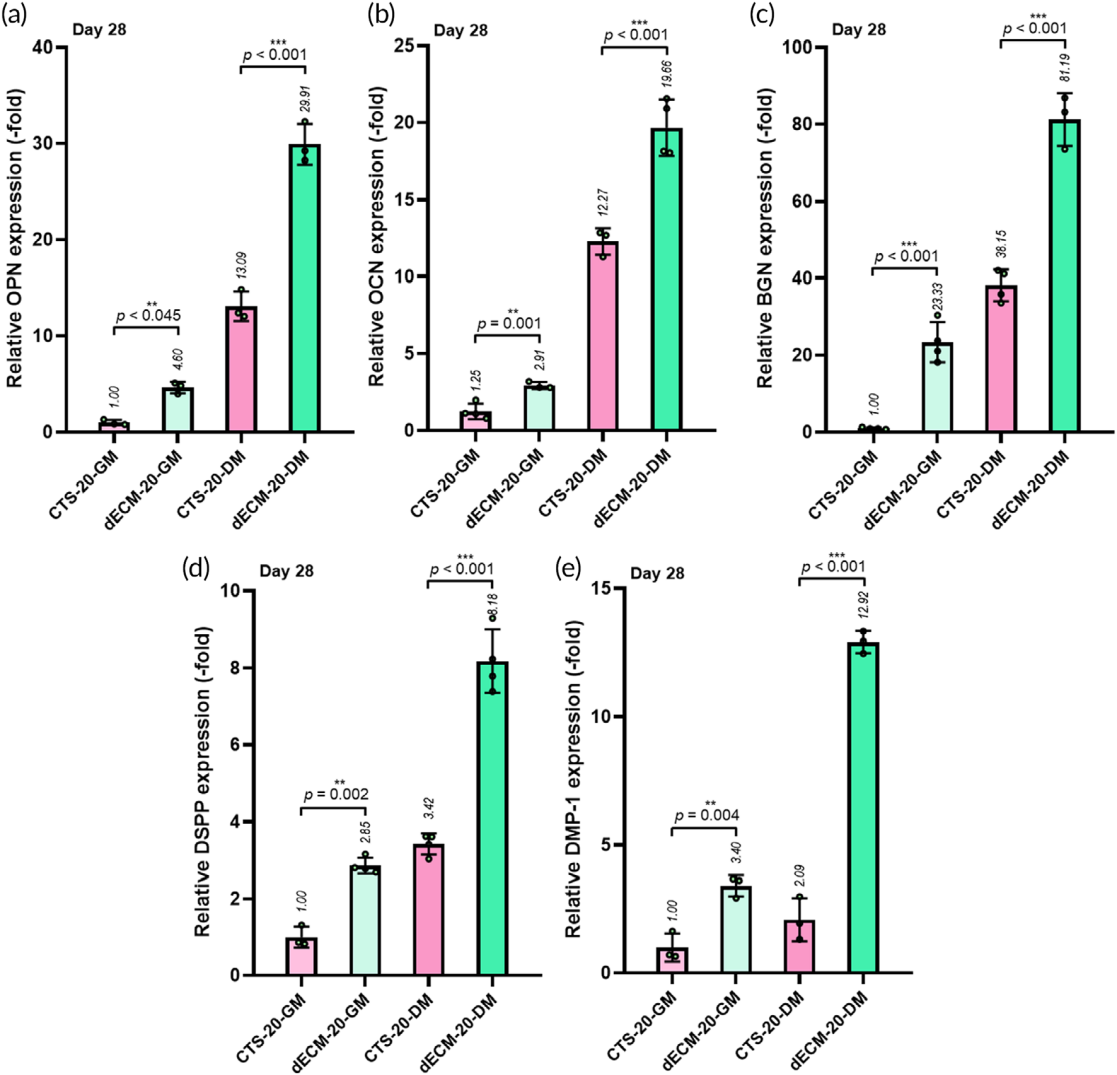
Gene expressions ((a) OPN, (b) OCN, (c) BGN, (d) DSPP, and (e) DMP‐1) of DPSCs in in vitro cultured biocomposites (CTS‐20 and dECM‐20) at 28 days under GM and DM (*n* = 3)

To quantitatively measure various osteogenic (OPN, OCN, and BGN) and odontogenic (DSPP and DMP‐1) gene expressions at 28 days, we performed RT‐PCR assay on the printed CTS‐20 and dECM‐20 structures (Figure 7a‐e). The results were compared with those of the housekeeping gene GAPDH. From the results, a significant increase in the expression of all the genes was observed in the dECM‐20 structure and osteogenic DM. In particular, the increasing trend was independent of osteogenic and odontogenic differentiation, as evidenced by the immunostaining results. Furthermore, the results of gene expression clearly indicate that the bioactive components, bone‐derived dECM and β‐TCP, can not only provide appropriate osteogenic microenvironmental conditions but also afford well‐organized odontogenic differentiation in the printed hDPSC‐laden structures. In addition, the enhanced odontogenic differentiation trend of the dECM‐20 structure was accelerated when using the osteogenic DM.

Based on the results, this work indicates that a bioprinted composite using a bone‐dECM‐based bioink and β‐TCP can clearly encourage osteo/odontogenic differentiation of hDPSCs in vitro, independent of the usage of GM and a mineralization medium.

### 3.5 Ectopic hard tissue formation

**FIGURE 8:**
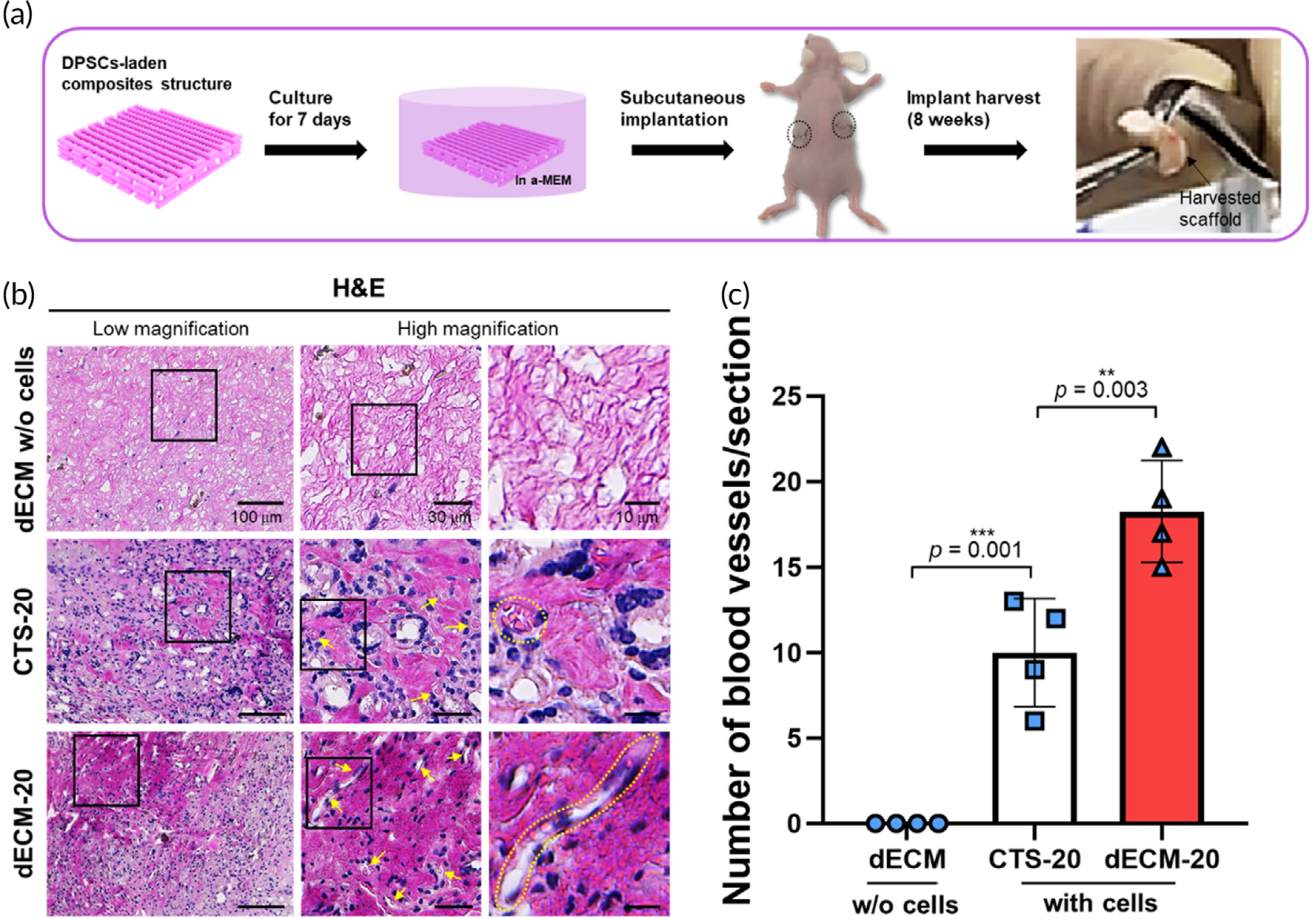
(a) In vivo subcutaneous implantation procedure of the bioprinted hDPSCs‐laden composite structures. (b) Histological examination for H&E (yellow arrows: blood vessels) staining of dECM without cells, CTS‐20, and dECM‐20 at 8 weeks after implantation. (c) Quantification of number of blood vessels per section (*n* = 4)

In this work, a comparative study of the hDPSCs‐laden structures (CTS‐20 and dECM‐20) and dECM structure without the cell was performed to assess ectopic bone‐formation after subcutaneous implantation in the dorsal area with the procedure, shown in Figure 8a. Experimental animals were healthy during the experimental period, indicating that any evidence for toxicity or side effects was not observed.

In general, the blood vessel formation in the implanted scaffold is a critical key component because the formation can specify efficient graft/host interactions and even signifies the stable cell‐survival for long term period.^33^ To evaluate the blood vessel formation of the implanted dECM without cells, CTS‐20, and dECM‐20, the implanted structures were histologically evaluated using hematoxylin and eosin (H&E) staining (Figure 8b). Figure 8c showed the vascularization of the implanted structures which were assessed using the H&E staining. As expected, vessel formation was plentiful in the dECM‐20 structure compared to that of the CTS‐20, which could be due to the synergistic effect of various vascular growth factors resided in the dECM‐bioink and the laden stem cells.

**FIGURE 9:**
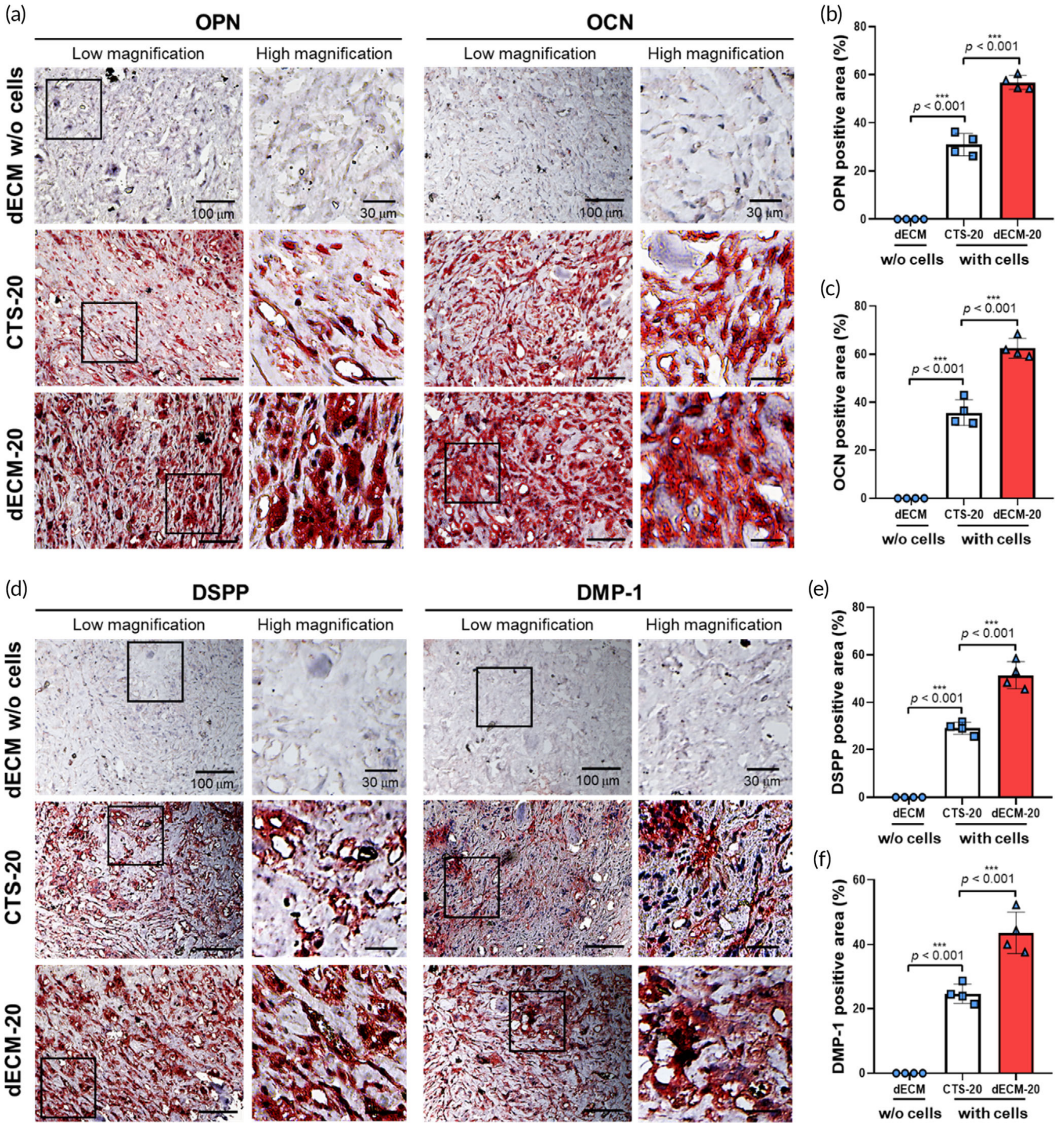
(a) Immunohistochemistry examination for OPN and OCN of dECM without cells, CTS‐20, and dECM‐20 at 8 weeks after implantation. (b,c) Quantification of OPN and OCN positive area (%) (*n* = 4). (d) Immunohistochemistry examination for DSPP and DMP‐1 of dECM without cells, CTS‐20, and dECM‐20 at 8 weeks after implantation. (e,f) Quantification of DSPP and DMP‐1 positive area (%) (*n* = 4)

After 8 weeks post‐implantation, an obvious de novo mineralization was observed in the hDPSCs‐laden structures (CTS‐20 and dECM‐20) compared to pure dECM structure, and the mineralization seemed much stronger in the dECM‐20. Osteogenic/odontogenic differentiation in the structures was also assessed using the markers of osteogenic (OPN and OCN) and odontogenic (DSPP and DMP‐1) differentiation. OPN and OCN, which are elaborated in osteogenic differentiation, showed strong signal in the cell‐laden structures (CTS‐20 and dECM‐20) (Figure 9a), specifically more in the dECM‐20 (Figure 9b‐c). Moreover, DSPP and DMP‐1, which are important factors in odontogenesis, were strongly expressed at 8 weeks, especially in the dECM‐20 structure (Figure 9d‐f). Based on the in vivo results, we confirmed that the structure using hDPSCs and bone‐derived dECM could obviously enhance both osteogenic/odontogenic‐mineralization and vascularization compared to collagen‐based cell‐laden biocomposite.

## 4 CONCLUSION

In this study, we fabricated a cell‐laden bone‐derived dECM/β‐TCP/hDPSC biocomposite, which was appropriately printed using various printing parameters for dental tissue engineering. To achieve the hDPSC‐laden composite, we formulated a bioink using various weight fractions of the bioceramic, with the goals of reasonable initial cell viability after printing and a mechanically stable 3D mesh structure. In addition, to achieve a biochemical cue to provide appropriate dental‐specific microcellular environmental conditions, we accommodated bone‐derived dECM, which has biological components similar to those of the dentin‐derived dECM. According to the results of various in vitro cellular activities and in vivo work, the dECM‐based biocomposite demonstrated meaningful cell viability and cell growth and even effectively accelerated the osteo/odontogenic differentiation of hDPSCs. Based on these results, we can conclude that the proposed 3D‐bioprinted biocomposite, supported with the biochemical cues derived from the dECM and β‐TCP, can serve as a potential dental tissue engineering material.

## AUTHOR CONTRIBUTIONS

**Dongyun Kim:** Data curation (equal); formal analysis (equal); investigation (equal); methodology (equal); writing – original draft (equal). **Hyeongjin Lee:** Data curation (equal); formal analysis (equal); investigation (equal); methodology (equal); validation (equal); writing – original draft (equal). **Geum‐Hwa Lee:** Investigation (equal); methodology (equal); validation (equal). **The‐Hiep Hoang:** Investigation (equal); methodology (equal). **Hyung‐Ryong Kim:** Funding acquisition (equal); methodology (equal); project administration (equal); resources (equal); supervision (equal). **Geun Hyung Kim:** Conceptualization (lead); data curation (equal); formal analysis (equal); funding acquisition (equal); methodology (lead); project administration (lead); supervision (lead); writing – review and editing (lead).

## CONFLICT OF INTEREST

The authors have declared that no competing interest exists.

### PEER REVIEW

The peer review history for this article is available at https://publons.com/publon/10.1002/btm2.10317.

## Supporting information


Data S1

## Data Availability

The data that support the findings of this study are available from the corresponding author upon reasonable request.
