# Supplementary material for: Fabrication of bone‐derived decellularized extracellular matrix/ceramic‐based biocomposites and their osteo/odontogenic differentiation ability for dentin regeneration
Source: Bioeng Transl Med. 2022 Apr 5;7(3):e10317. doi: 10.1002/btm2.10317 (PMC9472025; doi:10.1002/btm2.10317)
Supplement: Supplementary file 1 — Data S1 [file BTM2-7-e10317-s001.docx]

**Supplementary Information**

**Fabrication of** **Bone-derived decellularized extracellular matrix/Ceramic-Based Biocomposites and Their Osteo/Odontogenic Differentiation Ability for Dentin Regeneration**

*Dongyun Kim,^1,a^ Hyeongjin Lee,^1,a^ Geum-Hwa Lee,^2^ The-Hiep Hoang,^2^ Hyung-Ryong Kim,^3,*^ and GeunHyung Kim^1,4,*^*

^1^Department of Biomechatronic Engineering, College of Biotechnology and Bioengineering, Sungkyunkwan University (SKKU), Suwon, 16419, Republic of Korea

^2^Non-Clinical Evaluation Center, Biomedical Research Institute, Jeonbuk National University Hospital, Jeonju, Jeonbuk, 54907, Republic of Korea

^3^Department of Pharmacology, College of Dentistry, Jeonbuk National University, Jeonju 54896, Republic of Korea

^4^Biomedical Institute for Convergence at SKKU (BICS), Sungkyunkwan University, Suwon, Republic of Korea

^a^The authors contributed equally.

^*^Corresponding authors: Professor GeunHyung Kim, E-mail: [gkimbme@skku.edu](mailto:gkimbme@skku.edu).

Professor Hyung-Ryong Kim. E-mail: hrkimdp@gmail.com.

Competing Interests: The authors have declared that no competing interest exists.

**Supplementary Figures**

**
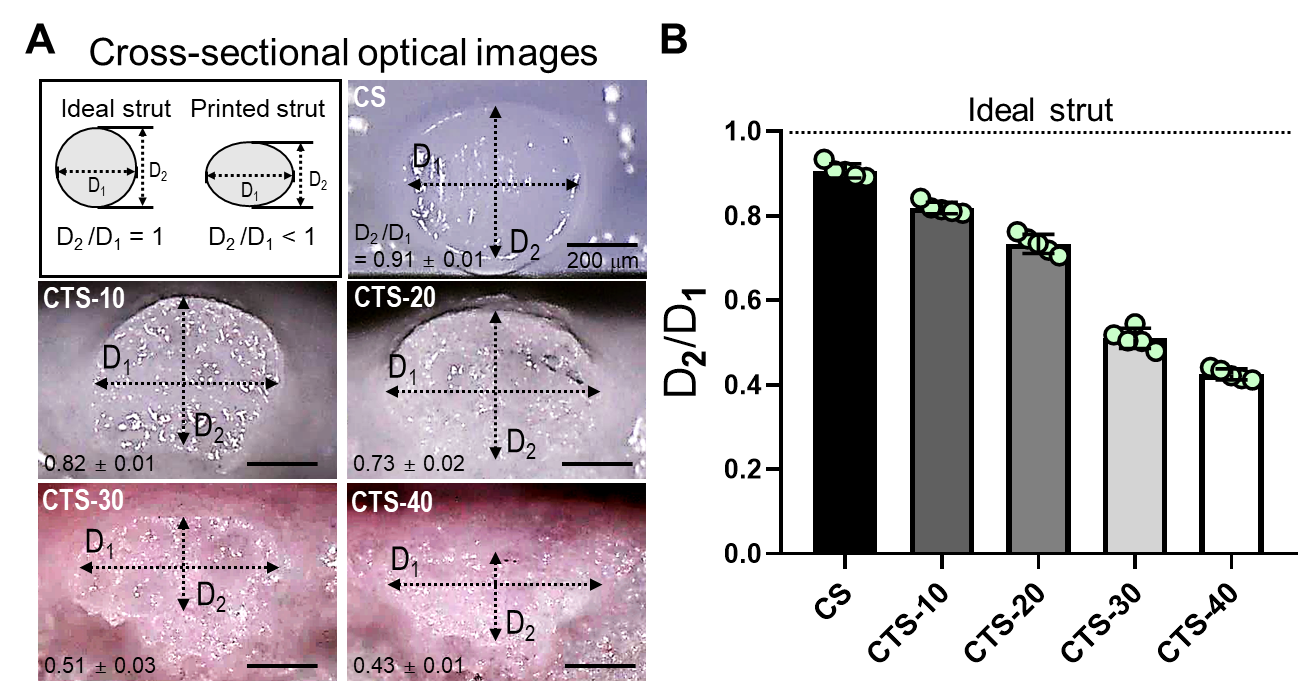
**

**Supplementary Figure S1** (a) Cross-sectional optical images for various weight fractions of β-TCP and (b) D_2_/D_1_ values of printed biocomposites.


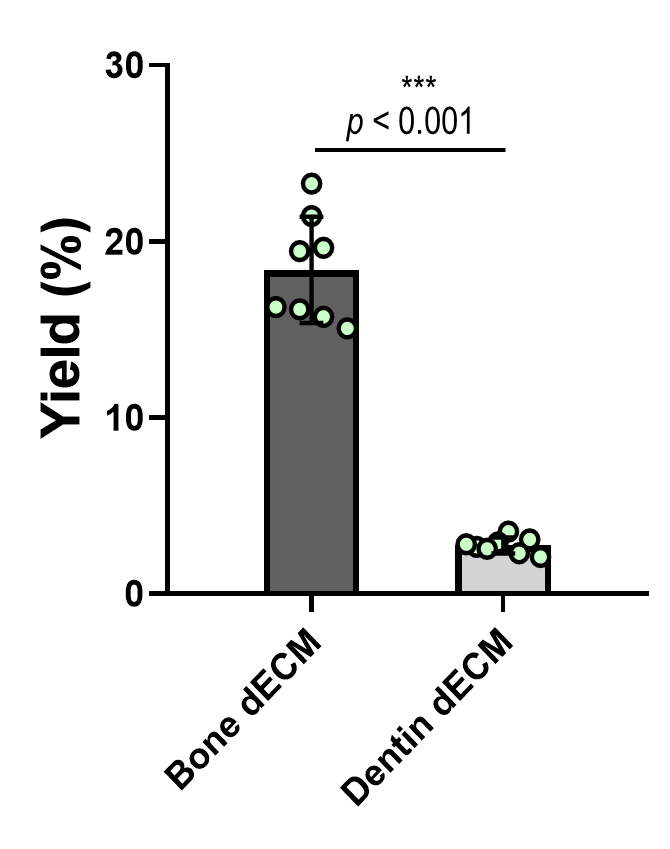


**Supplementary Figure S2.** Extraction yield rate of bone-derived dECM and dentin-derived dECM after demineralization and decellularization process from native tissues.

**
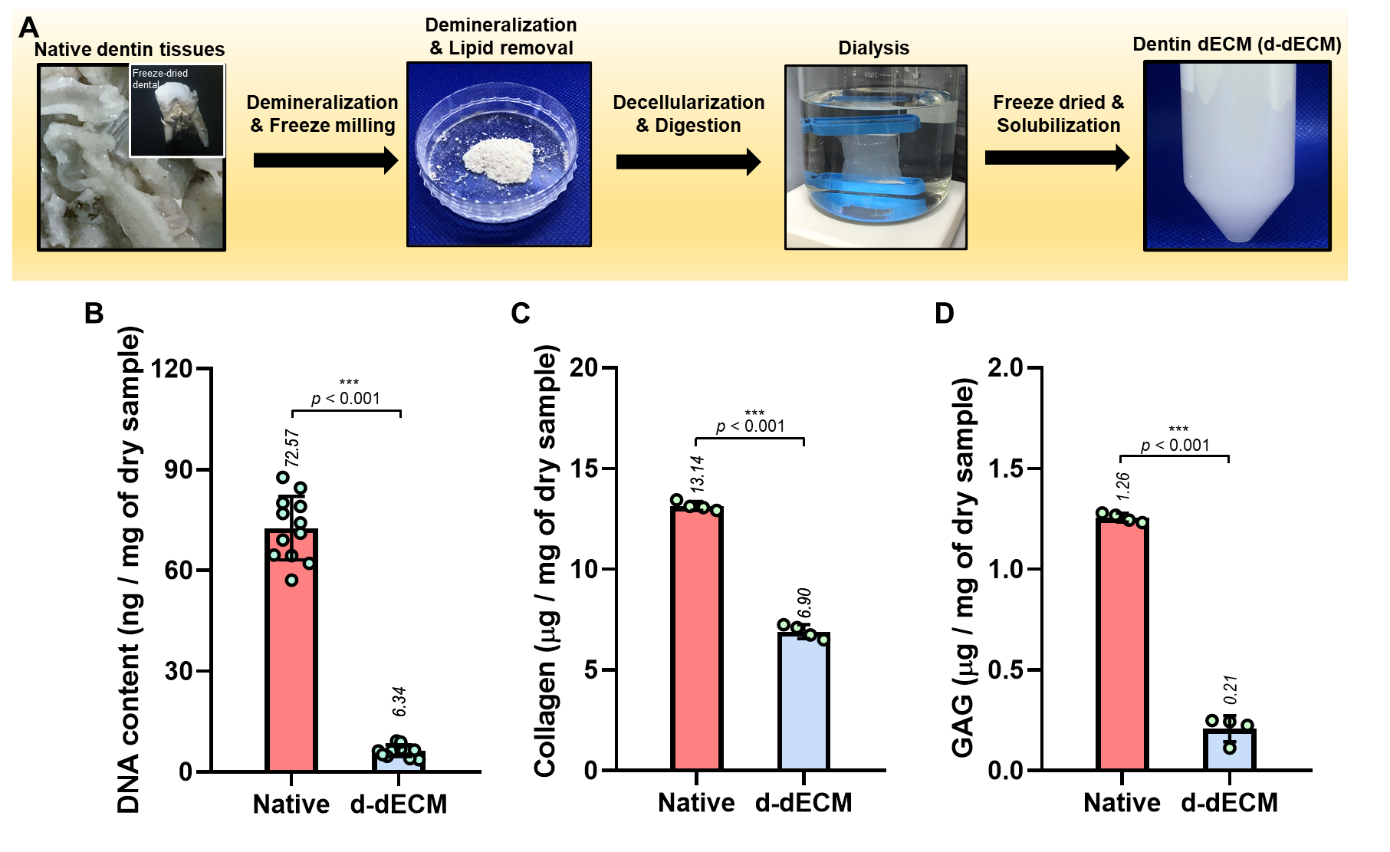
**

**Supplementary Figure 3.** (a) Schematic of the decellularization process using bovine-dentin-tissue and (b) DNA, (c) collagen, and (d) GAG contents for native tissue and dentin-dECM (d-dECM).


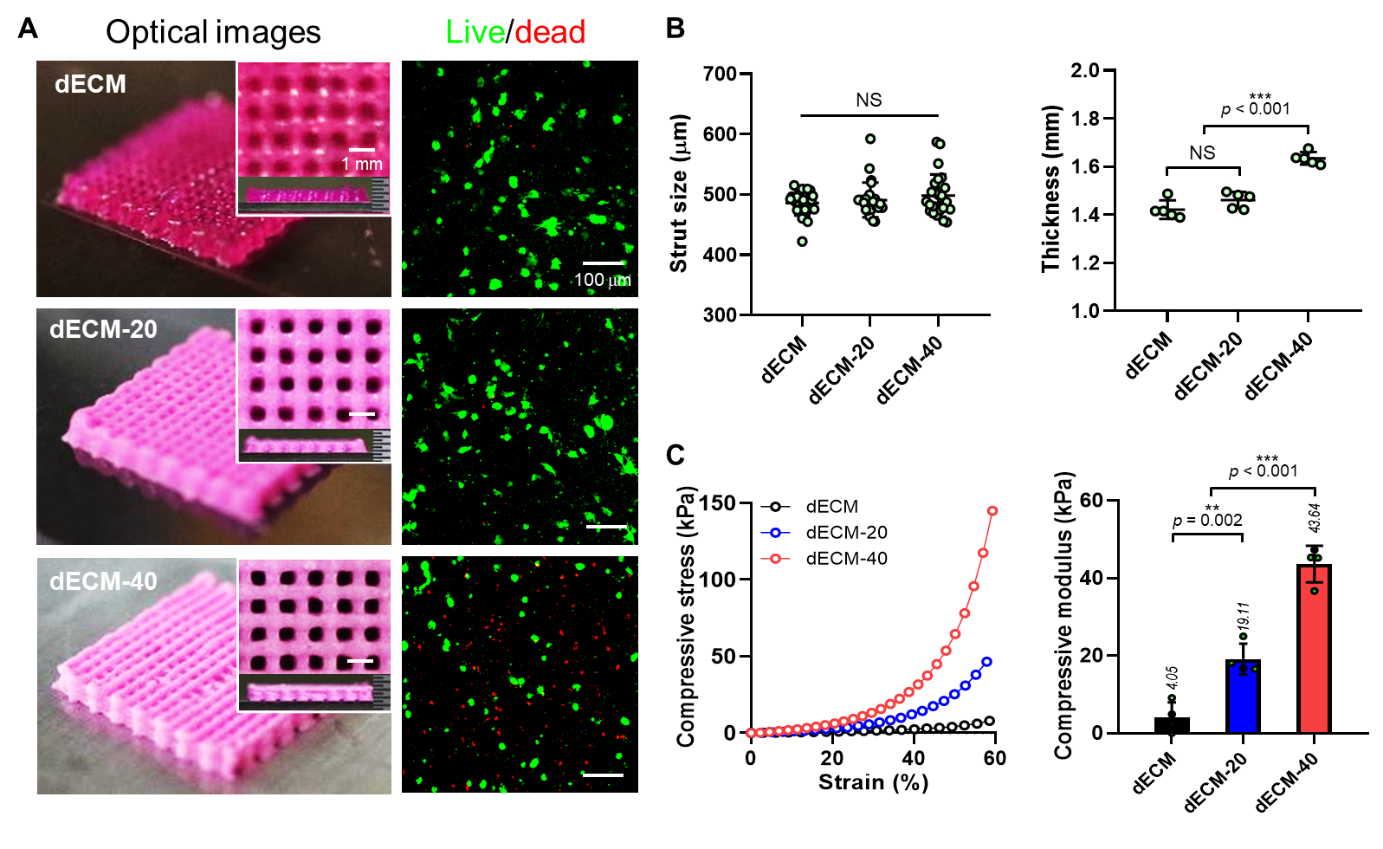


**Supplementary Figure 4.** (a) Optical and live/dead images of dECM-based biocomposites with various weight fractions of β-TCP (0, 20, and 40 wt%). (b) Geometrical sizes (strut and thickness) and (c) compressive stress–strain curves of the printed constructs.


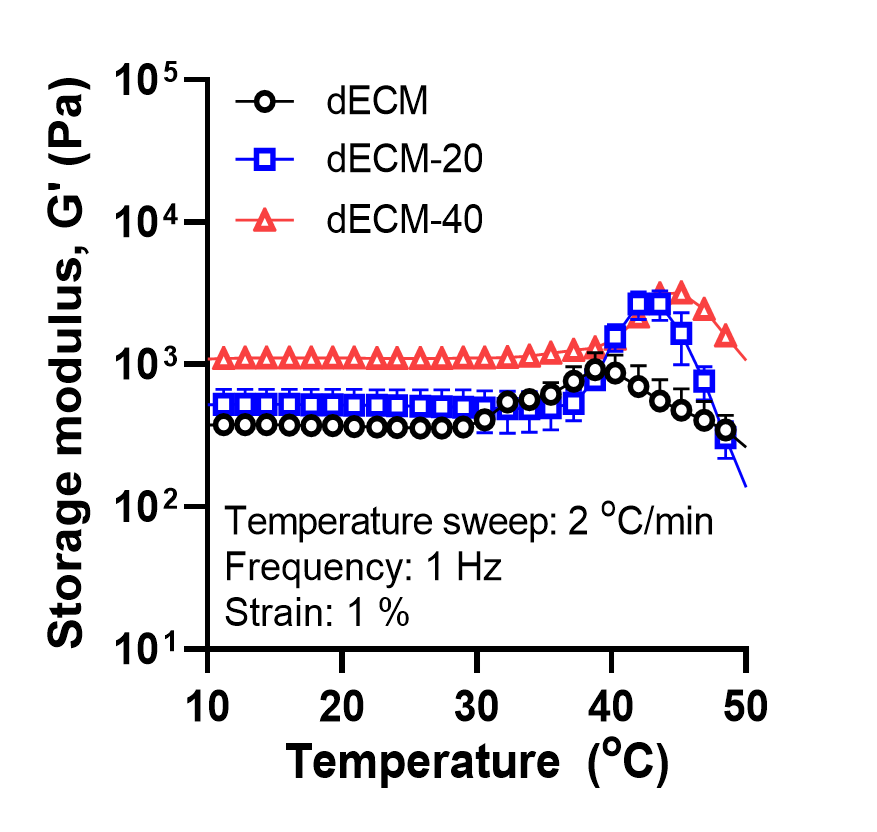


**Supplementary Figure 5.** Comparison of storage modulus values of dECM, dECM-20, and dECM-40 under temperature sweep.
